# Exploring senior nursing students’ perspectives on humanizing care for patients in isolation: a qualitative study

**DOI:** 10.3389/fmed.2026.1785546

**Published:** 2026-05-07

**Authors:** Júlio Belo Fernandes, Cidália Castro, Aida Simões, Diana Vareta, Inês Barros, Luiza Benatti, Steven Hall, Sónia Fernandes

**Affiliations:** 1 Egas Moniz Center for Interdisciplinary Research (CiiEM), Egas Moniz School of Health & Science, Caparica, Almada, Portugal; 2 Nurs* Lab, Caparica, Almada, Portugal; 3 Department of Nursing, Hospital de Santa Maria, Centro Hospitalar Universitário Lisboa Norte, Lisbon, Portugal; 4 Department of Nursing, Centro Hospitalar Barreiro Montijo, Barreiro, Portugal; 5 Faculty of Nursing, University of Alberta, Edmonton, AB, Canada

**Keywords:** humanized care, inpatient care, isolation units, nursing care, nursing theory, theory of human caring

## Abstract

**Background:**

Isolation units play a vital role in controlling disease transmission and ensuring the safety of both patients and healthcare professionals. However, the experience of isolation can lead to feelings of loneliness and anxiety that negatively affect patients’ wellbeing and recovery. Given this, making care more humanized in these settings should be a key priority in healthcare. This study explores nursing students’ perspectives on interventions that contribute to humanizing care for patients in isolation units.

**Methods:**

A theory-informed qualitative descriptive study was conducted with 19 senior nursing students completing clinical placements in isolation units across five hospitals. Interviews were transcribed and analyzed using Braun and Clarke’s six-step thematic analysis. Watson’s Theory of Human Caring was used as a sensitizing framework to support data interpretation.

**Results:**

The study identified several nursing interventions that enhance the humanization of patient care in isolation units. These interventions were classified into two main dimensions based on Watson’s theory. The *instrumental dimension* included interventions focused on symptom management, physical comfort, infection prevention and control, continuity of care, and discharge planning. The *expressive dimension* encompassed strategies directed at establishing a therapeutic relationship, individualized care planning, emotional and psychosocial support, relaxation and entertainment, patient empowerment, and family involvement.

**Conclusion:**

Categorizing these interventions within the framework of the Theory of Human Caring provides valuable insight into various approaches to humanizing care in isolation units. These findings can contribute to the development of patient-centered care models, encouraging healthcare professionals to deliver more comprehensive and compassionate care that addresses the holistic needs of isolated patients.

## 1 Introduction

The humanization of healthcare is an approach that aims to ensure dignity, respect, and empathy in patient care, especially for those in vulnerable and isolated conditions (1). There is growing awareness of the need to humanize nursing care for patients in isolation units (2, 3). This awareness was heightened during the COVID-19 pandemic and is further underscored by the ongoing global shortage of nurses and its impact on the quality and humanization of care (4, 5).

In isolation units, interactions with healthcare professionals become even more crucial for patients’ emotional and psychological support. However, contact with healthcare professionals is often limited, and most interactions are with nurses, who remain the primary point of care within isolation units. The global nursing shortage intensifies these challenges and is driven by workforce aging, rising patient needs, and insufficient training resources (4–6). The nursing shortage may further constrain the time and attention nurses can offer, compromising the humanization of care, particularly for patients in isolation (7).

Isolation units in healthcare organizations decrease the spread of communicable diseases and protect patients and healthcare workers. A patient may be isolated in a single room, in a multi-bedded area, or in a cohort of patients with the same infection. These units usually have strict infection control measures like personal protective equipment (PPE), isolation protocols, limited staff access, and restricted visits from family and friends (8). In isolation units, the use of PPE is essential to protect healthcare providers and prevent the transmission of infections (8). However, this equipment can create a physical barrier between nurses and patients, limiting opportunities for direct contact and touch, which are essential to effective communication and professional recognition, which are core elements of humanized care. Although these measures are important for patient safety, they can also make the patients feel lonely and undervalued, which can affect their mental health (9). These effects on mental health can contribute to increased levels of anxiety, depression, and a reduced sense of wellbeing, potentially hindering recovery and overall patient outcomes (10, 11).

By impeding interactions with patients, the use of PPE and other measures implemented in isolation units may compromise the delivery of truly humanized care, distancing professionals from the emotional and relational dimensions of patient support. This can significantly impact the patient’s emotional wellbeing and overall care experience (12). Addressing these challenges is therefore essential to ensure that infection control measures do not undermine the humanization of care.

Nurses play a key role in providing humanized care and are well-positioned to mitigate the adverse effects of isolation by offering physical, emotional, and psychological support (2). However, when there is a shortage of nurses or an increased workload, they may be forced to group interventions and reduce the time spent with each patient. This limits opportunities to build therapeutic relationships and deliver personalized care (13, 14). As a result, interactions may become rushed, communication may suffer, and emotional support may be lacking, leaving patients feeling anxious and neglected and compromising both their experience and clinical outcomes (15–17).

In this context, it is also important to consider the perspectives of nursing students as future professionals. Nursing education increasingly emphasizes person-centered care, empathy, and reflective practice, positioning students to critically engage with the humanization of care during their clinical training. Through their placements, including in isolation settings, students both observe and participate in care delivery, often identifying discrepancies between theoretical principles and clinical practice. This positions them as valuable contributors to understanding how humanized care can be supported and strengthened in real-world contexts.

Therefore, this study aimed to explore nursing students perspectives on interventions that support the humanization of care for patients in isolation units.

### 1.1 Theoretical framework

The theoretical framework we chose to guide this study was Watson’s Theory of Human Caring (18). Watson’s (18) theory describes that the delivery of humanized care is shaped by five factors: (1) patients ‘ personal life experiences; (2) their reactions to health issues and illnesses; (3) the dynamic interplay between the environment and the individual; (4) the collaboration among care providers; and (5) nurses’ comprehensive understanding of the care procedure. The theory posits that experiences are gained through self-awareness and recognizing power boundaries within the caring relationship between the nurse and the patient (19). This relationship relies on an interactive process, encompassing a meaningful connection between caregivers and recipients of care, which extends beyond mere tangible objectives to engage with the patient’s emotional and subjective world effectively (19).

Humanized care in isolation units encompasses various aspects that priorities patient-centeredness, communication, and emotional support. It involves acknowledging and addressing patients’ fears, concerns, and emotional needs while ensuring their physical wellbeing (2, 20). Humanized care also recognizes the importance of involving patients in decision-making, promoting autonomy, and fostering a sense of control amidst their isolation (2).

## 2 Materials and methods

The overarching research question guiding this study was: “What are nursing students’ perspectives on interventions that contribute to the humanization of care for patients in isolation units?” Our methods are reported herein in accordance with the Consolidated Criteria for Reporting Qualitative Research (COREQ) checklist (21).

### 2.1 Study design

This study used a theory-informed qualitative descriptive design (22) to explore senior nursing students’ perspectives on the interventions that humanize care for patients in isolation units. While grounded in participants’ experiences, the study was guided by Watson’s Theory of Human Caring as a sensitizing framework to support data interpretation. The study was approved by the Egas Moniz Ethical Committee (reference number 946).

### 2.2 Population and recruitment

The study population consisted of nursing students recruited from one nursing school in the region of Lisbon and Tagus Valley, Portugal. We used a non-probabilistic convenience sample, where nursing students were invited to participate in the study through their institutional email addresses.

The criteria for participating in the study were:

1. Senior undergraduate nursing students (in Year 4 of a 4-year baccalaureate nursing program).
2. Experienced a clinical placement in isolation units and/or delivered nursing care to patients in isolation rooms.

Senior nursing students were selected as participants due to their recent and direct exposure to clinical practice in isolation units during their placements. As trainees transitioning into professional roles, they are uniquely positioned to reflect both on observed practices and on emerging professional values related to humanized care. Their perspectives provide insight into how humanizing practices are perceived, learned, and enacted in contemporary clinical settings.

### 2.3 Data collection procedures

A research team member contacted via telephone the participants who registered their interest to present the research project, its aims, the concepts under study, and the importance of the participant’s collaboration. This was followed by an email where we provided written information regarding the research project to allow participants to reflect on and analyze the concepts before deciding on participating in the study.

All participants signed a consent form before the interviews. The interviews were performed individually and in person on the nursing school campus. During the interviews, no additional individuals were present apart from the interviewer and the participant. A nursing lecturer with a PhD in nursing science and experience in conducting qualitative interviews conducted the interviews. The interviewer had no pre-existing relationship with the participants. Interviews lasted approximately 40 min and were audio-recorded for later transcription.

The interview guide included questions to gather participants’ perspectives regarding nursing interventions that humanize care for patients in isolation units. We used open-ended questions with probes. The participants were asked questions such as: “Tell me about any interventions you can use to humanize care for patients in isolation units?”; “How can nurses humanize care for patients in isolation units?”; “Are there any interventions that were not available that patients could have benefited from?” Probes included: “Can you tell me more about that?” and “Please describe the impact of that nursing intervention.”

The interview guide was piloted among an initial four nursing students and experts in qualitative research who determined it to be explicit, objective, and helpful in retrieving the required information.

### 2.4 Data analysis

Researchers transcribed the interviews verbatim into a Microsoft Word document. Each participant’s transcription received a unique code number to de-identify the data. Participants were offered the opportunity to review their de-identified interview transcripts to provide any commentary or further clarification that may have improved data accuracy. The data analysis was executed concurrently with data collection.

Two researchers independently conducted a thematic analysis following Braun and Clarke’s six-step approach (23), supported by the QDA Miner Lite database. Discrepancies were resolved through discussion and consensus; when consensus could not be reached, a third researcher was consulted. Although the analysis was informed by Watson’s Theory of Human Caring (19), the coding process remained grounded in the data to ensure that participants’ perspectives were not constrained by the theoretical framework.

1. Two researchers immersed themselves in the data by thoroughly reading and rereading it, which allowed them to become acquainted with the interview data and form initial impressions.
2. They then generated initial codes inductively by identifying and labeling meaningful units of data, capturing the essence of participants’ accounts.
3. The researchers conducted an in-depth analysis by grouping codes with shared patterns and developing preliminary themes. These themes were subsequently interpreted in light of Watson’s theoretical dimensions, which were used as a sensitizing framework rather than as predefined categories.
4. The identified themes were reviewed and refined to ensure accuracy and alignment with the data, assessing the internal coherence of each theme and its relevance to the research objectives. Clear and concise descriptions were developed for each theme, accompanied by meaningful names that captured their essence and significance.
5. A cohesive narrative was then constructed, presenting the analysis with detailed explanations of each theme, supported by illustrative quotes. The report was written to be firmly grounded in the data, aligning with the tenets of qualitative description (22).
6. Finally, two other research team members assessed the analysis and confirmed the identified themes.

To illustrate the coding pathway, an example is provided in Table 1.

**TABLE 1:** Sample of coding pathway.

Data excerpt	Code	Subthemes	Theme
“Assigning a dedicated nurse to each patient helps build a closer, more personal connection.”	Continuity of care supports relational closeness	Continuity of care and discharge planning	Instrumental dimension of caring

### 2.5 Data saturation

We chose to focus on the richness of the selected cases rather than on sample size, as recommended in previous literature on qualitative methods (24, 25). Data collection and analysis were conducted concurrently, allowing for ongoing assessment of data adequacy. Data saturation was considered reached when no new codes or themes emerged from the data. This point was identified during the later stages of data collection and was confirmed after three consecutive interviews yielded no new information.

### 2.6 Trustworthiness

To ensure the trustworthiness of the data, we followed the practices recommended by Nowell et al. (26). The lead researcher approached eligible participants to establish rapport and explain the significance of the study, thereby ensuring trustworthiness. Face-to-face interviews were scheduled at times that were convenient for the participants, allowing them sufficient time to openly share their feelings and experiences.

The research team consisted of nurse academics with experience in qualitative research and a shared interest in humanized and person-centered care. This theoretical orientation was acknowledged as a potential influence on data interpretation. Although the interviewer had no pre-existing relationship with the participants, it is recognized that participants were recruited from the same academic institution, which may have introduced perceived power dynamics. To mitigate this, participation was voluntary, confidentiality was emphasized, and participants were reassured that their academic standing would not be affected by their decision to participate or by their responses.

Thorough discussions were held during data analysis between the research team. These discussions also supported reflexivity by encouraging critical reflection on emerging interpretations, underlying assumptions about humanized care, and potential influences of the researchers’ professional backgrounds. Although formal reflexive journaling was not undertaken, reflexive practices were embedded through ongoing dialogue and analytic scrutiny among the research team.

Considering that cultural, social, and religious factors may influence participants’ perceptions of humanized care, we ensured transferability by carefully following participants’ responses during the interviews to provide comprehensive descriptions of their feelings and experiences. In addition, we provided quotes from participants within this manuscript to further illustrate the transferability of our findings.

We also ensured the study’s reliability by maintaining a logical, traceable research process and documenting every step of the decision-making process. An audit trail was maintained throughout the research process, documenting coding decisions, theme development, and analytical changes. Finally, an external team of qualitative research experts cross-checked their perceptions with the researchers to establish confirmability.

## 3 Results

Nineteen nursing students participated in the study. Most participants were female (84.2%), with a mean age of 23 years (range 21–40), and had experienced a clinical placement in an isolation unit within one of five hospitals.

We identified several nursing interventions that enable the humanization of care for isolated patients. These interventions have been categorized (Table 2) and are detailed herein.

**TABLE 2:** Nursing interventions that humanize care for patients in isolation units.

Themes	Subthemes
Instrumental dimension of caring	Symptoms management
	Physical comfort
	Infection prevention and control
	Continuity of care and discharge planning
Expressive dimension of caring	Establishing a therapeutic relationship
	Individualized care planning
	Emotional and psychosocial support
	Relaxation and entertainment
	Patient empowerment
	Family involvement

### 3.1 Instrumental dimension of caring

#### 3.1.1 Symptoms management

Participants’ reports focused on collecting the patient’s medical history and conducting a physical examination to gather relevant information about past conditions, assess overall physical health, identify potential issues, and detect any changes in their condition. One participant said, “We conducted a thorough initial assessment, including a detailed medical history to help us understand their specific health needs and tailor the care accordingly.”

Participants’ reports also emphasized that providing interventions to address patients’ specific physical symptoms is vital to promote humanizing care. These interventions aim to alleviate physical discomfort, promote healing, and enhance patients’ wellbeing. One participant highlighted the impact of these interventions, stating, “They [the staff nurses] perform interventions to address the patient’s physical symptoms. They provide pain relief or offer comfort measures to help alleviate any discomfort. This intervention shows them that we care about their wellbeing.” Another participant highlighted the importance of providing interventions to promote healing and recovery. For example, nurses actively contribute to the patient’s physical wellbeing through wound care, physical therapy, and mobility assistance.

#### 3.1.2 Physical comfort

Assisting patients with activities of daily living held significant importance in humanizing care for patients in isolation units. These interventions promote dignity, maintain hygiene, and provide a sense of normalcy in a challenging healthcare setting. One participant stated: “Assisting patients with personal hygiene goes beyond physical care. These interventions are the essence of caring; it is about recognizing the patient’s humanity and treating them respectfully.” In addition, nursing students assisting with activities of daily living allows the patients to maintain a sense of normalcy and routine. One participant stated, “Helping patients with the activities of daily living allows them to maintain a sense of independence and normalcy. That is a way to promote a more humanized care experience.”

Participants also focused on providing supplies to meet patients’ basic needs. These supplies ensure patients access essential comfort, hygiene, and wellbeing items. By fulfilling these basic needs, nurses contribute to a sense of dignity for isolated patients. One participant said, “Nurses question patients daily if they need any supplies to maintain their hygiene and comfort.” Another participant highlighted, “Providing patients with familiar items, we create an environment that feels more like home.”

#### 3.1.3 Infection prevention and control

Adhering to strict infection control protocols, including proper hand hygiene, wearing PPE, and maintaining a clean and sanitized environment, are nursing interventions that humanize care for isolated patients. These measures ensure patient safety and create a conducive healing environment. One participant stated, “Nurses follow infection control protocols to ensure the safety of patients. Their actions show that they prioritize maintaining a safe environment that fosters patients’ recovery.” In addition to implementing infection control measures, nurses educate patients and their families on these preventive measures. Participants considered that nurses provide comprehensive education on hand hygiene, proper use of PPE, and other infection prevention practices. They described that this educational approach goes beyond implementing measures; it promotes patient engagement and collaboration in maintaining a safe healthcare environment. By teaching patients and their families about these measures, nurses ensure that everyone involved is well-informed and actively contributes to infection prevention efforts.

#### 3.1.4 Continuity of care and discharge planning

Maintaining continuity of care is crucial for patients’ wellbeing and ensuring a more humanized care experience. Nurses establish a trusting relationship and promote a sense of familiarity and comfort by ensuring that patients receive consistent and coordinated care. This continuity allows for a holistic understanding of the patient’s needs, preferences, and goals, enabling personalized care. One participant emphasized that “Assigning a dedicated nurse to each patient helps build a closer, more personal connection. It makes understanding the patient’s needs and preferences easier while ensuring continuous care.”

For participants, discharge planning emerged as a fundamental component of humanized care, as it represents not just the end of an isolation period but the beginning of reintegration into everyday life. Nurses, working with the healthcare team, assess the patient’s physical, emotional, and social needs to develop a comprehensive plan for post-isolation care. By actively involving patients and their families in the discharge planning, nurses facilitate the transition to the post-isolation period.

### 3.2 Expressive dimension of caring

#### 3.2.1 Establishing a therapeutic relationship

Participants’ reports suggested that humanized care comes from a strong connection and understanding between nurses and patients, making it easier to provide personalized care and emotional support. “The foundation of humanized care lies in building a trusting and empathetic relationship. If a nurse is consistently assigned to a particular patient [in isolation], they can establish a therapeutic relationship. That way, nurses develop a deeper connection and gain insight into the patient’s needs.” Other participants focused on the use of therapeutic communication to create a supportive environment that addresses patients’ emotional needs and the need to develop interventions to overcome the barriers caused by PPE, such as staff identification cards with enlarged photos and names, to enable patients to see and connect with their assigned nurses.

#### 3.2.2 Individualized care planning

Participants highlighted the importance of involving patients in developing personalized care plans. They emphasized that, through collaboration, nurses can ensure that care aligns with each patient’s needs, preferences, values, and goals. This approach acknowledges the uniqueness of each patient and promotes a patient-centered care environment. Moreover, participants also pointed out the significance of involving patients in decision-making processes related to their treatment and care options. For them, nurses empower patients to make informed choices about their health and wellbeing by including them in these discussions. This approach recognizes the importance of patient autonomy and promotes a sense of ownership over their care journey. One participant stated, “We saw that involving patients in decision-making is crucial to humanizing care, as it allows them to be actively engaged in decision-making and more in control of their health.”

#### 3.2.3 Emotional and psychosocial support

Participants underscored the significance of assessing and addressing patients’ emotional and psychosocial needs. They described that by actively identifying feelings of anxiety, fear, and social isolation, nurses can tailor their care to provide emotional support, reassurance, and interventions that alleviate these distressing experiences.

Participants also stated that nurses provide patients with appropriate emotional support during bedside operations through non-verbal gestures, such as eye contact, gentle touch, and nods. These interventions can convey empathy, understanding, and emotional support, contributing to the humanization of care. In addition, participants emphasized the importance of offering psychoeducational counseling to guide patients to cope with their emotional challenges effectively. One participant stated, “Nurses are essential to help patients develop skills to navigate their emotional journey during isolation.”

Participants described strategies for facilitating communication between isolated patients and their families, especially when physical visitation is restricted. By encouraging and facilitating virtual communication through video calls, phone conversations, or messaging applications, nurses help patients maintain social connections and emotional support from their family members and friends. In addition, participants also emphasized the importance of providing information on available support services, such as counseling or spiritual care services within the hospital setting, to address patients’ emotional and spiritual needs in a holistic manner, thus aligning with the concept of humanized care.

#### 3.2.4 Relaxation and entertainment

This subtheme highlights the importance of providing holistic support to patients, focusing on their emotional, mental, and spiritual wellbeing. Participants described encouraging patients to perform relaxation techniques, such as guided imagery or deep breathing exercises, to reduce stress and promote overall wellbeing. These interventions aim to create a calming and supportive environment that helps patients cope with isolation and reduce anxiety. In addition, nurses can facilitate access to complementary therapies—when available within the healthcare setting—to enhance patients’ comfort and relaxation. One participant stated that “Interventions like music therapy and aromatherapy can help patients feel more comfortable and relaxed, offering a temporary escape from isolation.” Another participant emphasized the importance of providing entertainment and fitness activities to help alleviate feelings of loneliness and isolation.

#### 3.2.5 Patient empowerment

Participants highlighted nursing interventions that empower patients to take an active role in their care, promoting autonomy and wellbeing. One crucial intervention for patient empowerment is providing clear and understandable information regarding their condition, treatment options, and care plan. One participant stated, “Providing information was key to humanizing care. I observed that when patients clearly understood their condition and treatment options, they participated actively in the care decisions.”

The participants felt that empowering patients involves creating an environment where they feel comfortable asking questions and expressing their concerns. Nurses actively encourage patients to voice their questions and concerns to ensure their needs are heard, respected, and addressed. Participants reported that empowering patients involves supporting their decision-making process and respecting their autonomy. Therefore, nurses are vital in providing information, guidance, and support while honoring patients’ autonomy. “Supporting them in decision-making is important because it proves that nurses respect their autonomy. We valued patients’ choices, and by doing so, we promoted their empowerment.”

#### 3.2.6 Family involvement

Participants highlighted the importance of including and engaging family members in the care process, recognizing their emotional needs, and fostering effective communication. They described the need to create a supportive environment for family members to express their emotions and the importance of obtaining information about their expectations to promote family involvement. By actively engaging with family members and acknowledging their perspectives, nurses can ensure that care plans align with patients’ values and preferences. One participant highlighted, “In that unit, most nurses recognize the value of involving the family to ensure that the care provided aligns with the patient’s wishes. We need to know their expectations to humanize the care.”

Participants also focused on incorporating mobile phone-based social networking applications to allow patients to communicate with their family members. This technology allows for real-time interaction, sharing of information, and emotional support, overcoming physical barriers imposed by isolation measures. However, participants indicated that while social networking applications are an excellent tool for enabling isolated patients to connect with others, some patients with physical and/or mobility impairments faced challenges using this technology because they needed to familiarize themselves with smartphones or develop additional physical skills to operate them effectively.

Although participants identified a wide range of interventions to humanize care in isolation units, their accounts also revealed important tensions. Humanized care was described as both essential and challenging to sustain in contexts shaped by infection control requirements, use of PPE, and time constraints associated with clinical workload. These findings suggest that humanization in isolation settings is not only dependent on individual nursing actions, but is also influenced by structural and organizational conditions that may facilitate or limit relational care.

## 4 Discussion

The findings of this qualitative study shed light on the nursing interventions that contribute to the humanization of care for patients in isolation units. The study utilized interviews with senior nursing students to identify these interventions, which were then categorized according to the theory of human caring (19) into two main themes: the instrumental dimension of caring and the expressive dimension of caring.

The use of senior nursing students as participants offers a distinctive perspective on humanized care in isolation settings. As individuals in transition between academic learning and professional practice, students are uniquely positioned to observe, interpret, and reflect on care practices while simultaneously developing their own professional values. Their accounts not only reflect observed clinical behaviors but also reveal how humanizing practices are understood, internalized, and shaped during the process of professional socialization.

At the same time, their perspectives may be influenced by variations in clinical exposure, supervision, and levels of active participation in care, which can shape how humanized care is perceived and enacted (27, 28). This highlights the importance of considering experiential diversity when interpreting the findings and reinforces the need to support students in translating humanizing principles into practice within complex clinical environments.

The instrumental dimension of caring focuses on the practical and action-oriented interventions necessary to address the physical needs of patients in isolation (19). By effectively managing symptoms, ensuring physical comfort, preventing infections, and promoting continuity of care, nurses create an environment that supports patients’ overall wellbeing and meets patients’ specific physical health requirements in isolation. However, it is crucial to recognize that physical wellbeing alone is insufficient for genuinely humanizing care. The expressive dimension of caring complements instrumental interventions by emphasizing patient-centered care (19), which encompasses individualized care planning, psychosocial support, relaxation, entertainment, patient empowerment, and family involvement.

Integrating both dimensions of care is essential to provide comprehensive and humanized care to isolated patients. By incorporating both dimensions of care, nurses transition from a disease-focused approach to a more humanized care practice (2). However, the degree to which care addresses these dimensions and the extent to which it aligns with the humanizing features of each intervention remains to be determined. Therefore, it is essential to interpret instrumental and expressive interventions as two interconnected dimensions that cannot be separated but imply one another for care to be genuinely humanizing (19).

Humanized care involves holistic care considering patients’ physical, emotional, social, and spiritual elements. It requires a comprehensive understanding of the individual as a whole person (1, 21). To develop humanized nursing care, it is crucial to recognize that these dimensions are inseparable. They represent different facets of holistic nursing care and contribute to the humanization of care (1, 19). Therefore, nursing care must be planned and implemented to simultaneously address the instrumental and expressive aspects. By acknowledging and integrating both dimensions, nurses can provide care that addresses patients’ physical, emotional, spiritual and social needs.

While participants described a wide range of interventions to support humanized care, their accounts also reveal important tensions embedded in isolation settings. Infection prevention measures, including the use of PPE and restricted contact, are essential to ensure patient and staff safety; however, these same measures may limit opportunities for therapeutic presence, touch, and relational closeness. Humanized care in isolation contexts, therefore, emerges as a continuous negotiation between the need to protect and the need to connect.

The findings also highlight that the implementation of humanized care is shaped by structural and organizational constraints. Although participants emphasized the importance of individualized attention, emotional support, and continuity of care, these practices require time, adequate staffing, and supportive work environments. In contexts marked by increased workload and nurse shortages, the capacity to sustain relational and person-centered care may be compromised, suggesting that humanization is not solely dependent on individual professional competence, but also on institutional conditions.

In addition to Watson’s theoretical framework, these findings can also be interpreted through broader perspectives on person-centered care and relational practice. The emphasis on individualized care, patient involvement, and meaningful nurse–patient relationships aligns with person-centered care approaches, which highlight the importance of recognizing the person beyond the clinical condition. From this perspective, humanized care is not only an ethical or relational ideal but is also associated with improved patient experience, greater engagement in care, and more positive health outcomes, reinforcing its relevance in clinical practice. Furthermore, the tensions identified in this study resonate with relational ethics, where care is understood as a situated and negotiated practice shaped by context, vulnerability, and responsibility. From this perspective, humanized care in isolation settings can also be seen as involving forms of emotional labor, as nurses must continuously balance professional, emotional, and organizational demands while maintaining compassionate engagement with patients.

It is essential to acknowledge that nurses are utilizing new technologies to humanize care for isolated patients. However, participants’ reports highlighted that these technologies are more prevalent among younger patients, while more dependent patients may not engage with them as readily. Incorporating mobile phone-based social networking applications and other technological advancements can enhance communication and bridge the gap between isolated patients and their friends and relatives, thereby reducing feelings of social isolation (29, 30). With that being said, it is crucial to recognize that not all patients, especially those with limited physical or technological skills, can effectively use these tools. Nurses should dedicate time to help these patients connect virtually with their family members and friends, ensuring a supportive and inclusive environment (31). This may involve providing hands-on guidance on operating mobile-phone-based applications, facilitating video calls or virtual meetings, and addressing technical difficulties.

Allocating additional time for patients to connect with their families through these technological means demonstrates a commitment to humanizing care. Additionally, nurses can explore alternative means of communication that better suit the needs and preferences of less technologically inclined patients. This could involve facilitating phone calls or coordinating visits from family members following strict infection control protocols. By considering individual patient circumstances and preferences, nurses can ensure that all patients have the opportunity to maintain meaningful connections with their families, regardless of their technological proficiency.

At the same time, these strategies reveal a degree of ambivalence. While digital technologies can reduce social isolation and support emotional wellbeing, they may also inadvertently reinforce inequalities, particularly among patients with cognitive, physical, or technological limitations. This highlights the need to tailor interventions to individual capabilities and contexts, reinforcing that humanized care is inherently relational and situational rather than standardized.

### 4.1 Limitations

Some limitations exist with this study. Firstly, the study was conducted with interviews with students from only one nursing school and different schools of nursing may hold different philosophies, emphasizing different aspects of humanized care in their course deliveries. However, participants performed their clinical placements in different units from five hospital centers, meaning perspectives could be varied significantly. Therefore, we believe this diversity in experiences and perspectives from different hospitals helps to mitigate this limitation. Secondly, interpreting what constitutes a humanizing nursing intervention is subjective among the interviewed students due to different perspectives on what qualifies as a humanized nursing intervention. Lastly, there are some limitations regarding the practice implications of our findings. Although nurses can easily implement most of the interventions identified in the study, some may involve financial costs and additional resources beyond what is available in standard care settings. An increased amount of time would also need to be dedicated to individual patients on a nurse’s patient assignment. This can be problematic considering the current global shortage of nurses, limited hospital resources and poor patient ratios.

## 5 Conclusion

Overall, this study’s findings underscore the significance of nursing interventions in humanizing care for patients in isolation units. By categorizing the interventions into the instrumental and expressive dimensions of caring, this study highlights the holistic nature of nursing care, which encompasses both the physical and psychosocial aspects of patient wellbeing. Ultimately, our findings contribute to theoretical and practical knowledge regarding care provision in isolation units and provide valuable insights for nurses and other healthcare professionals to humanize their care in these settings.

## Data Availability

The raw data supporting the conclusions of this article will be made available by the authors, without undue reservation.
